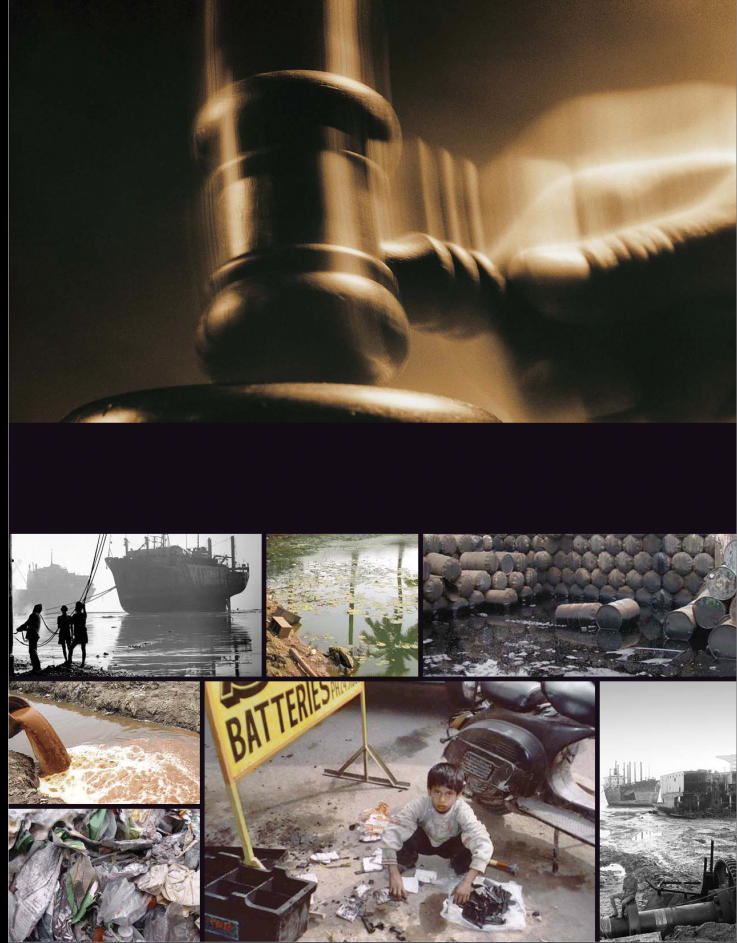# By Order of the Court: Environmental Cleanup in India

**DOI:** 10.1289/ehp.113-a394

**Published:** 2005-06

**Authors:** Dinesh C. Sharma

Metal scrap from New York’s World Trade Center towers. Live missiles and mortar shells from Iraq and Somalia. Used lead-acid batteries from Canada. Aging oil tankers and military carriers from Europe. This is just a little of the imported waste found in scrap yards and hazardous stockpiles across India. That’s in addition to large amounts of toxic wastes generated and dumped by local industries every day. Many industries dump sludge and effluents laden with heavy metals and persistent organic compounds in open areas, in rivers, and around residential areas, in gross violation of national laws. At some places, toxic dumps have contaminated soil and groundwater for decades, making communities around them sick. Industry’s near-total disregard for laws relating to hazardous waste, coupled with apathy and inaction by state agencies, has made the situation grim.

Today, more than 13,000 licensed industries generate about 4.4 million metric tons of hazardous waste every year, according to estimates from the Indian Ministry of Environment and Forests (MEF). This doesn’t include small-scale businesses such as backyard smelters. According to the ministry, the five states of Maharashtra, Gujarat, Tamil Nadu Karnataka and Andhra Pradesh generate about 80% of the waste in India. Unsound practices have caused widespread degradation of the environment and adverse health impacts on Indian communities and industrial workers.

Now, however, in a significant gesture, the Indian Supreme Court has taken up the challenge of forcing polluters and states to clean up these hazards. Helping the court in this task is a monitoring panel of scientists and concerned citizens.

## A Heightening of Awareness

Economic liberalization policies in the past 20 years or so have led to rapid growth in Indian industries. The production of petrochemicals, pesticides, pharmaceuticals, textiles, dyes, fertilizers, leather products, paint, and chlor-alkali has grown significantly. These industries produce wastes containing heavy metals, cyanides, pesticides, complex aromatic compounds (such as polychlorinated biphenyls), and other toxics. Several toxic waste hot spots—such as the industrial belt of Vapi and Vadodara in Gujarat, Thane-Belapur in Maharashtra, and Patancheru-Bollarm in Andhra Pradesh—developed in this period.

At the same time, India woke up to the dangerous realities of industrial hazards after the Bhopal disaster in 1984. The government enacted the Environment Act in 1986; under this legislation, the Hazardous Waste Rules were formulated in 1989.

These rules required each industry generating hazardous waste to obtain authorization from its state pollution control board. Boards, in turn, could issue authorization only after verifying that the industry had the facilities, technical capability, and equipment to safely handle hazardous waste. Industries were to deposit their hazardous waste in disposal sites set up by state governments and specifically designed to receive different kinds of waste. Significantly, the rules permitted the import of hazardous waste for processing or reuse as raw material.

But followup action such as creating secured landfills has come slowly. Fifteen states were given funds to identify landfill sites, but none were opened until 1997. Likewise, although India joined the Basel Convention in 1992, the nation’s hazardous waste rules were brought into compliance with convention stipulations only in 2000.

In the absence of disposal mechanisms permitted under the rules industries either stored wastes onsite or dumped them in the open. Temporary storage—permitted for 90 days under the 1989 rules—became permanent. It was hazardous waste anarchy.

In 1995, in response to a petition by a New Delhi–based nongovernmental organization (NGO) now known as the Research Foundation for Science, Technology, and Ecology, the Supreme Court asked relevant agencies for information on the amount of hazardous waste imported and generated domestically, as well as how it was being disposed of. But the state pollution control boards were not collecting data properly, so for two years, the MEF and the Central Pollution Control Board (CPCB; the entity that oversees the state boards) had no authentic data to provide. So the court convened a panel to investigate and make recommendations. Known as the High Powered Committee on Management of Hazardous Wastes (HPC), this panel submitted its final report in 2001.

## A Legacy of Toxic Wastes

The committee’s findings were grim. “Most industries used the opportunity [presented by the delay in constructing disposal sites] to discharge their hazardous waste in illegal dump sites outside industrial estates, along roadsides, in low-lying areas, along with municipal wastes, or even in river and canal pits,” observed the 2001 HPC report. The report further noted that “the authorities appeared to have ignored several warnings, reports, investigations, and studies that highlighted zones of ecological degradation due to indiscriminate dumping and disposal of hazardous waste.” The committee reported the existence of 80 illegal dumps in Andhra Pradesh and Gujarat alone. Satellite imagery is now being used to locate and confirm the extent of wastes strewn throughout the Thane region of Maharashtra.

Meanwhile, other findings began coming to light. In the Gorwa industrial area of the city of Vadodara, Hema Chemicals had been dumping 77,000 metric tons of highly carcinogenic hexavalent chromium waste over the past 20 years or so, according to the Gujarat Pollution Control Board. A 2001 study by the National Institute of Occupational Health of Ahmedabad, Health Surveillance of Workers Exposed to Chromium in a Chemical Industry, revealed blood chromium levels in exposed Hema employees to be more than twice as high as control subjects. No systematic studies have been carried out on nearby communities, but a local NGO, Paryavaran Surksha Samiti, claims that blood chromium levels in area residents also are high.

In a 1997 report, *Groundwater Quality in Kanpur, Status Sources and Control Measures*, the CPCB reported chromium concentrations 124–258 times higher than the Indian permissible limit in areas polluted by tanneries and companies making basic chrome sulphate. They also found high levels of several other contaminants such as mercury, arsenic, chloride, and lead. Although the polluted water is not fit even for irrigation, people continue to drink it as alternate supplies are not available, says Rakesh Jaiswal, executive secretary of the NGO Eco-Friends Society in Kanpur. The CPCB study found people blending chromium-rich sludge with coal ash to make a binding material for building. The contaminated sludge has also been used in road construction.

Ship-breaking is another source of toxicants. Ship-breaking activity at Alang-Sosiya has resulted in wastes containing heavy metals and petroleum hydrocarbons. For many years, most of the wastes have been dumped on the coast or burned in the open. Studies by the Central Salt and Marine Chemicals Research Institute have shown that wastes accumulate in the soil first and then migrate incrementally to the tidal zone, the subtidal zone, and finally to deep seawaters and into sediments. High levels of trace metals such as cobalt, nickel, copper, lead, and cadmium have been found in sediments. No formal studies have been done in Alang-Sosiya, but anecdotal evidence indicates sea creatures are dying off as a result of the pollution.

Still other threats are posed by lead. Field studies in Karnataka and Gujarat—conducted by the University of Cincinnati Department of Environmental Health and the National Referral Centre for Lead Poisoning in India, Bangalore—have indicated abnormally high environmental lead levels near lead smelters, lead-acid battery assembly units, service centers, and electronic soldering units. Thuppil Venkatesh, head of the referral center, says soils near battery dismantling and smelting units had lead levels up to 100,000 parts per million. Due to the pressure of growing urbanization, he says, people continue to live near such units.

Recycling of imported waste is a legal business in India, with all kinds of waste—from discarded electronics to cow dung—coming from more than 100 countries. Lists of allowable items have been developed (although companies have taken advantage of lax implementation at ports). Metal scrap, including dead ammunition, can be imported, but preshipment inspection was mandatory only for imports from war zones. However, when live missiles and bombs exploded in scrap yards near Delhi last year, killing 14 workers, the government changed the rules and made pre-shipment inspection necessary for all scrap imports. Old computers and other electronics waste is being sent to India for recycling under the aegis of charitable donations. Discarded lead-acid batteries can be imported only by licensed recyclers using safe technologies, but these batteries find their way into the unlicensed informal recycling market. The government finds it difficult to ban waste imports altogether because waste recycling provides employment to a large number of people.

## Judicial Activism

In recent years, in the face of these and other findings, the Supreme Court has spurred major environmental actions such as relocating polluting industries out of Delhi and replacing diesel with compressed natural gas in public transport. In so doing, the court has expanded the scope of “the right to life”—a concept enshrined in the constitution of India—to include the right to a clean and healthy environment.

“It is not as if the court is encroaching upon territories of legislature or [government]—it is only protecting citizens’ rights guaranteed under the constitution and various laws like the Environment Act,” points out Sanjay Parikh, a Supreme Court attorney representing the public interest in the hazardous waste case. “If the state does not fulfill its legal and constitutional obligations, then the court can direct it to do so.”

Under the Indian constitution, Parikh says, the Supreme Court’s directives are to be treated as law until the government enacts suitable legislation or changes existing regulations. This often happens in response to petitions filed by individuals or groups, but may also be initiated by the Supreme Court itself. At times, even informal complaints written on postcards and sent to the court have been treated as petitions, and proceedings initiated.

In the case of hazardous waste, the Supreme Court intervened because the government had signed the Basel Convention but failed to change the rules to check the import of hazardous waste. Parikh says it was the court’s intervention that led to regulatory mechanisms and procedures for the import, transport, storage, recycling, and final disposal of hazardous waste. The Supreme Court’s October 2003 final judgment on the 1995 petition set a detailed timetable for such actions as amending various sets of rules; reviewing lists of hazardous waste; setting up testing laboratories at ports to verify the content of declared hazardous waste; construction of secured landfills and treatment, storage, and disposal facilities (TSDFs); closure of industries violating rules; and disclosure of such information to communities.

As a result of the court’s intervention and the HPC’s recommendations, the MEF amended the 1989 rules in 2000 and 2003 to make them more stringent. Categorization of waste produced by different industrial processes as well as from imports has been further refined. A new list of 29 categories of hazardous waste completely banned for import and export has been added. The roles of different agencies have been clearly demarcated. New sets of rules for recycling and handling of used lead-acid batteries and plastic waste have also been codified.

Another set of amendments currently under way will introduce new measures such as punitive action for illegal imports and the allowance of re-exports after 30 days if imported wastes are in contravention of rules. (Currently, wastes cannot be re-exported after 30 days, so many exporters simply dump them at ports.) The list of banned wastes also is being further scrutinized.

## Committee Effort

In a rare gesture, the Supreme Court also constituted a committee to oversee implementation of its 2003 judgment (followup is usually left to the party receiving the judgment). The Supreme Court Monitoring Committee (SCMC) reports quarterly to the court on progress being made toward each of the points in the timetable.

The SCMC’s work has brought more hazardous waste skeletons to light. One example is Travancore Titanium Products at Thiruvananthapuram, found to have been operating close to a beach for several years without an effluent treatment plant. “Effluent of pH less than one and temperature more than fifty degrees centigrade is being discharged into the open sea in violation of every conceivable law,” the committee noted in its March 2005 report.

The SCMC ordered the unit to close, but the company was granted a stay in the Kerala High Court, giving it until 2006 to set up an effluent treatment plant. The SCMC has pleaded to the Supreme Court that such stays granted by high courts prevent the committee from carrying out its mandate. On 9 May 2005 the Supreme Court directed that no high court or any other authority shall interfere with directions of the Supreme Court given in the October 2003 judgment.

The committee has ordered the closure of several other industries and is applying the “polluter pays” principle for cleaning up the mess. The amount of hexavalent chromium waste dumped by Hema and two other companies—Golden Chemicals and Tamil Nadu Chromates—has been estimated to be 250,000 metric tons. The SCMC called chromium pollution by Hema Chemicals a case of “deliberate poisoning of communities with toxic wastes, contaminating water, soil, and air.” Hema has been asked to pay about US$3.9 million for remediation of the surrounding area. In another instance, the SCMC has directed costs of mercury decontamination to be recovered from Hindustan Lever, which owned a thermometer factory at Kodaikanal. In Bhopal, action groups have demanded that Dow Chemical—with whom Union Carbide merged in 2001—pay for the cleanup of toxic dumps at the closed Union Carbide plant. But the SCMC has not taken a final view on this.

## Other Progress

In Kanpur, the CPCB is partnering, of its own volition, with a consortium of Indian research institutes and New York’s nonprofit Blacksmith Institute for remediation of groundwater polluted by hexavalent chromium. “We will chart migration pathways of pollutants that have traveled both vertically and horizontally over all these years, using mathematical modeling. This could offer a model for cleaning up similar groundwater pollution sites and will help communities access clean water,” says R.K. Singh, a CPCB scientist.

Twenty-one ship-breaking units in Alang-Sosiya have been closed, and citations have been issued to 11 others for improper handling of wastes. A TSDF site for ship-breaking waste has been identified. Meanwhile, wastes are being transported to another facility in Ahmedabad.

In line with the Supreme Court judgment and the HPC report, the SCMC says ship-breaking activity can continue with proper safeguards such as decontamination in the exporting country itself. Other stakeholders disagree with this decision, however. “In our view, ships that come [into India] with waste oil, asbestos, poly-chlorinated biphenyls, and radioactive material are a violation of the Basel Convention,” says Ramapati Kumar, toxics campaigner of Greenpeace India.

In Gujarat, 13 industries have set up their own TSDFs, while 6 other facilities have been set up for use by clusters of industries. Battery manufacturers have started buying back their used products, to avoid their going to backyard smelters. Large users of batteries such as railways now auction their old batteries only to registered recyclers.

“We see some signs of change like establishment of secured landfills and TSDFs in states that were earlier refusing to do so, display of information on hazardous waste at factory gates, initiation of projects to clean up dumps, and stern action to close down violators,” says Gopalkrishnan Thyagarajan, chairman of the SCMC. Overall, he says, there is greater acceptance by industry of the committee’s authority, and industry’s attitude is changing. At many places, local watchdog panels have been set up with scientists, prominent citizens, NGO representatives, and pollution control officials as members.

Public reaction to court actions has been very favorable, at least over the long term. The phaseout of diesel vehicles from Delhi is a case in point. Bus owners cursed the courts at first, and the common people, too, suffered through the growing pains of the transition period. But today the average city dweller is thankful to the Supreme Court for its role in improving Delhi’s air quality.

## An Active Future for the Court?

Despite all these efforts, the overall scenario remains serious. A large number of units are still operating without authorization in several states, and illegal dumping of wastes continues in Maharashtra, Tamil Nadu, Gujarat, and Delhi, the SCMC told the Supreme Court in its March 2005 quarterly report. The committee has asked state pollution control boards to hire detective agencies and encourage whistle-blowers to report such practices.

Preparation of inventories of hazardous waste generated and illegal dumps is running behind schedule. In the absence of reliable inventories of wastes, few efforts are made to use tools such as environmental impact assessments, risk assessments, or health impact assessments for addressing hazardous waste problems, says Suneel Pandey, a fellow at the Centre for Environmental Studies at The Energy and Resources Institute of New Delhi. “Although the government recognizes the localized nature of hazardous waste generators, and large dumps are being identified, we need to quantify and characterize the volume of waste residues,” he says. “Also, since the growth of the industrial sector is dynamic, there is a need to constantly upgrade this waste inventory in order to develop suitable management strategies.”

Necessary rules and regulations have gradually been put in place for the import, handling, transport, and safe disposal of hazardous waste, but central and state pollution control boards tasked with implementing them remain weak. “The need is to strengthen these boards and the existing institutional base so that enforcement can be made sustainable. After all, a monitoring committee can’t have an endless tenure,” says K.P. Nyati, head of the environment management division of the Confederation of Indian Industry.

Also, the rules do not provide any incentive to industry for waste reduction or minimization. So companies are reluctant to adopt such measures, says Pandey. Moreover, he says, in the absence of standards for the cleanup of contaminated sites and limits for the disposal of wastes on land, polluters are not legally bound to clean up a site unless ordered by judicial intervention to do so.

For the foreseeable future, it seems likely that such judicial intervention will continue. The active role played by the Indian judiciary in the past two decades has redefined its place in India’s society. Courts are increasingly being viewed not just as a mechanism to settle disputes, but as a platform to protect citizens’ rights and to undo wrongs committed by the government. In a move that other governments will certainly take note of, the Indian Supreme Court has taken a keen interest in environment-related matters, and its judgments have impacted society at large.

## Figures and Tables

**Figure:**